# Preconditioning Local Injection of Activated Platelet-Rich Plasma Increases Angiogenesis, VEGF Levels, and Viability of Modified McFarlane Flap in Diabetes-Induced Rats

**DOI:** 10.1055/a-2317-4520

**Published:** 2024-06-13

**Authors:** Jenisa Amanda Sandiarini Kamayana, Agus Roy Rusly Hariantana Hamid, Tjokorda Gde Bagus Mahadewa, I. Gusti Putu Hendra Sanjaya, I. Made Darmajaya, I. Gusti Ayu Sri Mahendra Dewi

**Affiliations:** 1 Division of Plastic, Reconstructive and Aesthetic Surgery, Department of Surgery, Udayana University, Bali, Indonesia; 2 Department of Neurosurgery, Udayana University, Bali, Indonesia; 3 Division of Paediatric Surgery, Department of Surgery, Udayana University, Bali, Indonesia; 4 Department of Anatomical Pathology, Udayana University, Bali, Indonesia

**Keywords:** platelet-rich plasma, diabetes mellitus, VEGF, angiogenesis, random skin flap

## Abstract

**Background** The risk of flap necrosis in tissue reconstruction surgery is elevated in patients with vascular disorders, such as diabetes mellitus. Chronic hyperglycemia causes endothelial cell dysfunction and increases inflammatory process, causing vascular insufficiency. Platelet-rich plasma (PRP) contains high levels of platelets, growth factors, and fibrinogens. Its regenerative properties spark interest in supporting flap survival in relation to diabetic complications.

**Methods** Thirty Wistar rats were divided into three groups. The first group included diabetic rats without PRP injection, which underwent flap procedure. The second group included diabetes-induced rats receiving PRP subcutaneous injection 1 day prior to flap procedure. The third group included nondiabetic rats receiving PRP injection 1 day prior to flap procedure. Flap tissue samples were taken on the seventh day to measure vascular endothelial growth factor (VEGF) levels using enzyme-linked immunosorbent assay method; angiogenesis and collagen density were measured from histopathology examination, and flap viability was analyzed using digital measurements.

**Results** Analysis showed that flap viability, angiogenesis, and VEGF levels were significantly higher in the PRP-injected diabetic rats compared with diabetic rats that did not receive PRP. The levels of VEGF, angiogenesis, and viability of flaps in diabetic rats given PRP did not differ significantly compared with nondiabetic rats that received PRP.

**Conclusion** Flap preconditioning through local injection of activated PRP enhances flap viability, VEGF levels and angiogenesis, in random skin flaps in diabetic rats, to the level where it does not differ significantly to nondiabetic rats that were given PRP.

## Introduction

Tissue reconstruction with pedicled vascularized tissue represents a pivotal approach for addressing various conditions, including trauma, posttumor excision, and chronic wounds such as diabetic ulcers. The risk of flap necrosis in tissue reconstruction procedures is notably higher in patients with vascular disorders, as exemplified by diabetes mellitus. Diabetes mellitus is a metabolic disorder characterized by persistent hyperglycemia stemming from impaired insulin secretion, insulin resistance, or a combination thereof. Chronic hyperglycemia precipitates macrovascular and microvascular blood vessel dysfunction, resulting in endothelial cell structural and functional impairment, as well as disruption of cell regeneration and proliferation, increased inflammation, and escalated susceptibility to wound infection and eventually culminating in the risk of sepsis. 1 Diabetic patients exhibit a 2.3-fold higher likelihood of free flap failure in comparison to nondiabetic patients. 2 Rapid advances in regenerative medicine have expanded the potential of several therapies to accelerate wound healing in diabetes, such as the use of exogenous growth factors, cytokines, and stem cell therapy; nevertheless, the accessibility of these approaches is limited by their elevated cost implications.

Platelet-rich plasma (PRP) constitutes a blood plasma product enriched with high levels of platelets, growth factors, and fibrinogens. It has recently generated considerable attention within the field of regenerative therapy due to its natural sourcing, simple processing method, and cost-effectiveness. It contains various growth factors and cytokines that support the acceleration of tissue healing. 3 The impact of PRP preparation on skin flap survival is extensively recognized. Chai et al (2019) revealed that application of autologous PRP gel onto skin flap enhances flap viability by reducing inflammation and promoting tissue growth. 4 The regenerative properties of PRP on skin flaps is similarly evident in other pathological conditions such as in irradiated tissue. 5 However, there are limited data on the effects of PRP application on skin flaps among diabetic patients. Consequently, further investigation is necessary to estimate the potential beneficial effects of PRP on skin flap viability within the diabetic population.

This study aims to investigate the effects of activated PRP in the form of flap preconditioning through local injection on skin flap viability in diabetic rats.

## Methods

### Animal Management

Ethical clearance for this study was obtained from The Research Ethics Committee of the pertinent institution, with approval number 3135/UN14.2.2.VII.14/LT/2022. Thirty male Wistar rats were randomly divided into three groups, each comprising 10 rats. The rats were 8 to 12 weeks of age, weighing between 200 and 250 g. Throughout preoperative and postoperative periods, individual rats were housed in separate fiberglass cages with sufficient ventilation, maintaining standard living conditions of 50 to 55% humidity and room temperature ranging from 25 to 30 °C, with a 12-hour day/night cycle.

### Study Group

The Wistar rats in this study were categorized into three study groups. The first group was injected with streptozotocin (STZ) to induce diabetes and subsequently underwent a modified McFarlane flap procedure on the dorsal area without PRP injection. The second group was injected with STZ to induce diabetes and underwent a modified McFarlane flap procedure with local PRP injection performed a day before flap placement. Meanwhile, the third group underwent a modified McFarlane flap procedure with PRP local injection a day before flap placement without prior induction of diabetes. All animals across the study groups received similar postoperative interventions.

### Diabetes Mellitus Induction

Induction of diabetes in rats for the first and second groups was done by administering 10% fructose in drinking water ad libitum for a duration of 2 weeks, followed by a single intraperitoneal injection of STZ. 6 On the day of injection, rats were subjected to a 12-hour fasting period, followed by injection of single dose of 40 mg/kg STZ (ChemCruz®, TE, Huissen, the Netherlands) diluted in 0.01 molecule/L citrate buffer with pH of 4.5. The rats' blood glucose levels were assessed 24 hours post-STZ injection, following which the rats were left untreated for 6 weeks. Blood glucose levels were measured 1 day prior to the flap procedure. The cutoff blood glucose value to determine the success of diabetes induction was above 200 mg/mL, indicative of moderate to severe diabetes.

### Platelet-Rich Plasma Preparation

Blood collection, from donor Wistar rats, obtained as much as 4 mL each from the retro-orbital sinus, into vacuum blood tubes containing 0.5 mL of 3.8% sodium citrate as an anticoagulant, followed by double centrifugation processing. The first centrifugation was performed at 1,300 rotations per minute (rpm) for 10 minutes, resulting in the separation of the plasma on the top layer, the buffy coat underneath, and erythrocytes at the bottom. The plasma layer and buffy coat were transferred into another centrifugation tube devoid of anticoagulant, and subjected to a second centrifugation at 2,000 rpm for 10 minutes. This resulted in PRP at the bottom layer and platelet-poor plasma (PPP) at the top layer. The PPP layer was transferred into a separate tube, while 100 μL of PRP was left in the tube. To facilitate PRP injection, PRP resuspension was conducted using 200 μL of PPP, resulting in a PRP suspension with a volume of 300 μL. The minimum platelet concentration needed in the PRP in our study aligns with previous studies, which is more than 200,000 to 300,000 platelets/mL. 7 8 The double centrifugation process in our study yielded PRP with an average platelet concentration of 2,450,000 platelets/mm ^3^ . Afterward, immediately prior to utilization, the PRP suspension was activated with 33 μL of 10% calcium gluconate (OGB Dexa®, Banten, Indonesia). Following activation, the PRP preparation was used immediately before initiation of the clotting process.

### Local Injection of Platelet-Rich Plasma

Twenty-four hours before the flap procedure, activated PRP was subcutaneously injected into the dorsum of rats within the intended flap area in the second and third groups. The rats were anesthetized using a combination of ketamine (75 mg/kg) and xylazine (10 mg/kg). Injection of PRP in each rat was given with a total volume of 600 μL, administered across eight injection points.

### Flap Surgical Procedure

A modification of the McFarlane flap was used in this study, a random skin flap that receives vascularization from the subdermal plexus. The flap procedure was performed under anesthesia in all three groups; however in the second and third groups, it was performed 24 hours after flap preconditioning with PRP injection. After identifying the anatomical landmarks, including the superior margin of the iliac crest, the spinous process, and the lower scapular angle, flaps were designed with dimensions of 3 × 10 cm. The flap was incised and elevated, ensuring inclusion of the panniculus carnosus, a layer of striated muscle within the subcutaneous tissue. Finally, the flap was sutured back to its original position with 4/0 nylon. 9 Following this, the wound was kept clean using a transparent dressing to mitigate infection risk. Data collection was conducted on the seventh day postoperation. The rats were anesthetized, and digital photography was captured from a distance of 30 cm. The viable area of the flap was digitally calculated using ImageJ® (Media Cybernetics, Silver Spring, MD), subtracting area of distal necrosis from the total flap area, stated in percentage. Tissue samples were collected from the central flap area for vascular endothelial growth factor (VEGF) level measurement via enzyme-linked immunosorbent assay (ELISA) method. Histopathological examination for this study was performed by a single, blinded pathologist with over 10 years of experience in the field. Tissue samples were preserved in 10% buffered formalin, embedded in paraffin blocks, sliced and stained with hematoxylin and eosin. Under light microscopy, observations were made of characteristics between each group, including evaluation of angiogenesis and collagen density. Following tissue sample collection, the rats were euthanized using a high dose of ketamine and xylazine, and cremated through incineration in the appropriate environment.

### Statistical Analysis

Quantitative data obtained from the experiment were analyzed using one-way analysis of variance and its corresponding post hoc least significant difference test for normally distributed data. Non-normally distributed data were analyzed using the Kruskal–Wallis test and Mann–Whitney post hoc test. Statistical significance was set at a *p* -value <0.05. Flap viability and VEGF levels data were normally distributed and reported in mean and standard deviation. Angiogenesis and collagen density data were non-normally distributed and thus presented as median and interquartile range (IQR).

## Results

### Animal Model Serum Glucose and Body Weight

Following STZ injection, rats in the first and second groups were left untreated for 6 weeks. STZ injection resulted in an elevation of blood glucose levels in these two groups to >200 mg/dL, signifying successful induction of diabetes ( Table 1 ). The average blood glucose level on the day preceding the flap elevation procedure was 522.20 ± 123.87 mg/dL in the first group and 597.70 ± 7.27 mg/dL in the second group. Conversely, blood glucose levels in the third group, which were not subjected to diabetes induction, averaged 108.10 ± 16.64 mg/dL. Diabetic rats exhibited characteristics and symptoms consistent with diabetes, along with observations of thinner skin and reduced subcutaneous fat tissue.

**Table 1:** Blood glucose level of rats 1 day prior to flap elevation

Group	Blood glucose (mg/dL)
DM	522.20 ± 123.87
DM + PRP	597.70 ± 7.27
Normal + PRP	108.10 ± 16.64

Abbreviations: DM, diabetes mellitus; PRP, platelet-rich plasma.

### Skin Flap Viability

**Fig. 1:**
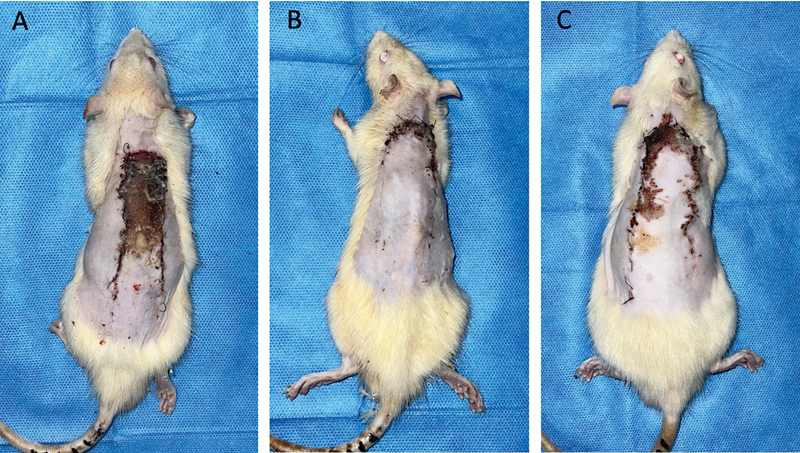
Viability of modified McFarlane flap in Wistar rats in each group. ( **A** ) Diabetic rats; ( **B** ) diabetic rats with PRP injection; ( **C** ) nondiabetic rats with PRP injection. PRP, platelet-rich plasma.

**Fig. 2:**
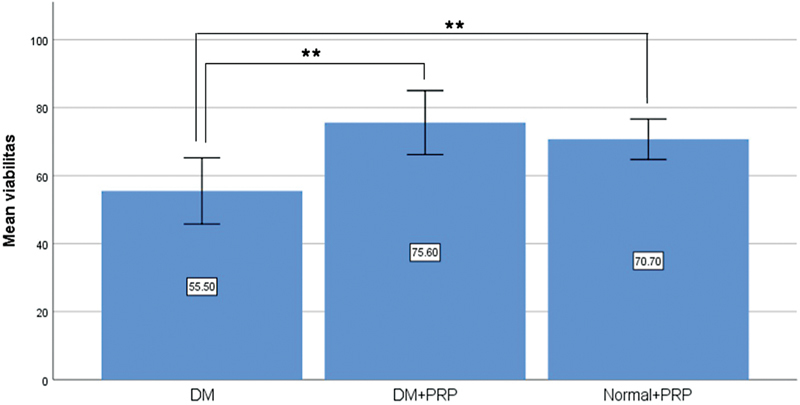
Viability of modified McFarlane flaps in each group (** *p* < 0.01). DM, diabetes mellitus; PRP, platelet-rich plasma.

The mean percentage of viable flap area on the seventh day postoperatively was higher in diabetic rats injected with PRP compared with diabetic rats not receiving PRP, measuring 75.60 ± 13.17 and 55.50 ± 13.62%, respectively ( Fig. 1 ). This difference was proved statistically significant ( *p* = 0.001; Fig. 2 ). Meanwhile the mean percentage of viable flap area in nondiabetic rats injected with PRP was 70.70 ± 8.31%, demonstrating no significant difference compared with PRP-injected diabetic rats ( *p* = 0.367).

### Vascular Endothelial Growth Factor Levels

**Fig. 3:**
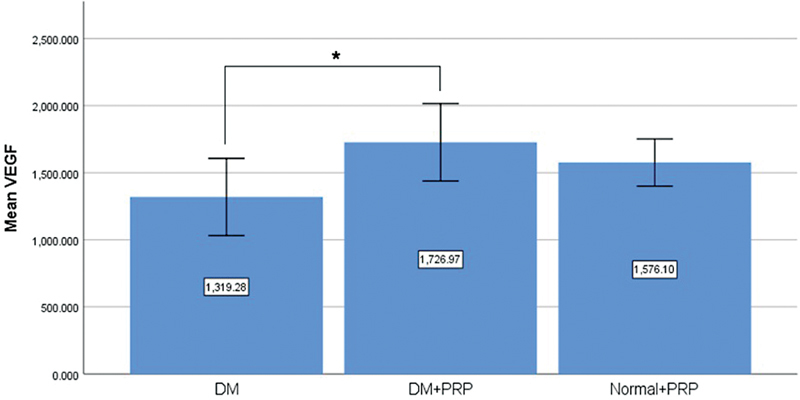
Levels of VEGF in flap tissue of each rat group (* *p* < 0.05). DM, diabetes mellitus; PRP, platelet-rich plasma; VEGF, vascular endothelial growth factor.

To evaluate whether PRP treatment influences VEGF levels in the flap, tissue samples taken from central area of the flap were obtained on day 7, and VEGF levels were quantified using the ELISA method (BT Laboratory, Zhejiang, China). Findings indicated that the mean VEGF level in flaps of diabetic rats injected with PRP was significantly elevated compared with flaps of diabetic rats without PRP injection, measuring 1,726.97 ± 403.61 and 1,319.28 ± 402.06 ng/L, respectively ( *p* = 0.017; Fig. 3 ). Moreover, the mean VEGF level in nondiabetic rats injected with PRP was 1,576.10 ± 246.00 ng/L, demonstrating no significant difference compared with PRP-injected diabetic rats ( *p* = 0.355).

### Histopathological Evaluation

**Fig. 4:**
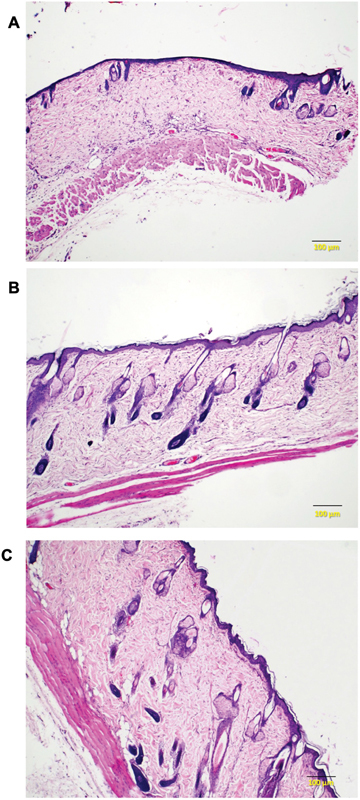
Histological images show differences in the thickness of the skin flap tissue and the integumentary system in the three groups (H&E, ×40). ( **A** ) Diabetic rats; ( **B** ) diabetic rats with PRP injection; ( **C** ) nondiabetic rats with PRP injection. PRP, platelet-rich plasma.

Tissue samples were fixed in 10% buffered formalin, embedded in paraffin blocks, sliced, and stained with hematoxylin and eosin. Under light microscopy, observations were made of characteristics across each group. The dermis and epidermis layers in the first group appeared thinner, with markedly reduced skin appendages, in contrast to the second and third groups. Additionally, adipocytes in the subcutaneous layers of the diabetic groups were diminished in comparison to the nondiabetic group ( Fig. 4 ).

**Fig. 5:**
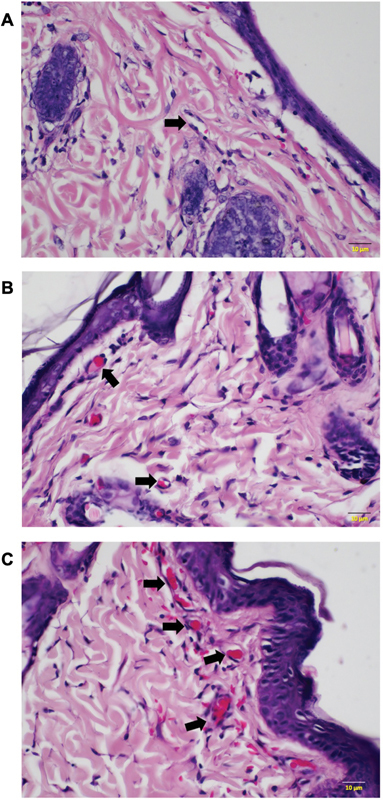
Histological images for evaluation of angiogenesis (H&E, ×400). Angiogenesis was higher in diabetic rats with PRP injection ( **B** ), compared with in diabetic rats without PRP injection ( **A** ). Nondiabetic rats with PRP injection showed the highest number of capillaries in the flap ( **C** ). PRP, platelet-rich plasma.

**Fig. 6:**
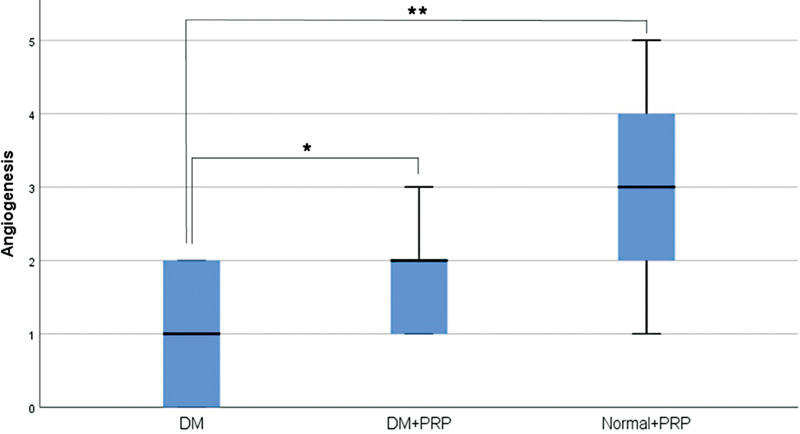
Angiogenesis in flap tissues in each group (* *p* < 0.05, ** *p* < 0.01). DM, diabetes mellitus; PRP, platelet-rich plasma.

Flap tissue samples were examined for blood vessel count, indicative of flap angiogenesis ( Fig. 5 ). On the seventh day postoperatively, it was revealed that angiogenesis in diabetic rats receiving PRP injection was higher compared with diabetic rats without PRP injection, with median values of 2.00 (IQR 1) and 1.00 (IQR 2), respectively. This difference was statistically significant ( *p* = 0.035; Fig. 6 ). Similar to flap viability and VEGF levels, the increase of angiogenesis in diabetic rats treated with PRP reached a value that was not significantly different to nondiabetic rats receiving PRP, at 3.00 (IQR 2; *p* = 0.063).

**Fig. 7:**
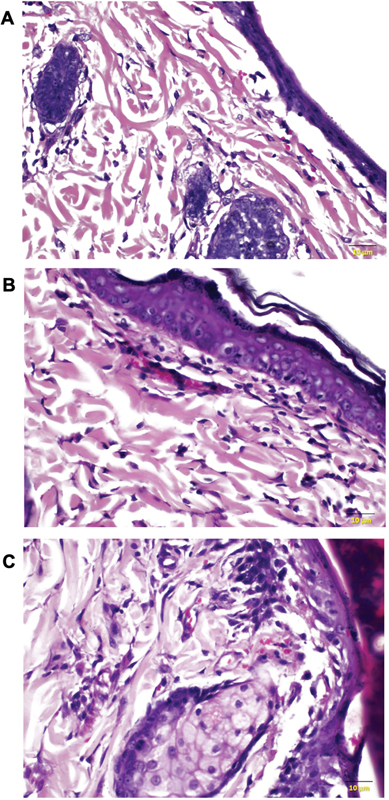
Histological images for evaluation of collagen density (H&E, ×400). Diabetic rats without PRP injection showed the lowest collagen density with disorganization of collagen fibers ( **A** ). Diabetic rats with PRP injection showed higher collagen density and improved collagen fiber organization ( **B** ). Better organization of collagen fibers with the most dense deposition seen in the group of nondiabetic rats with PRP injection ( **C** ). PRP, platelet-rich plasma.

**Fig. 8:**
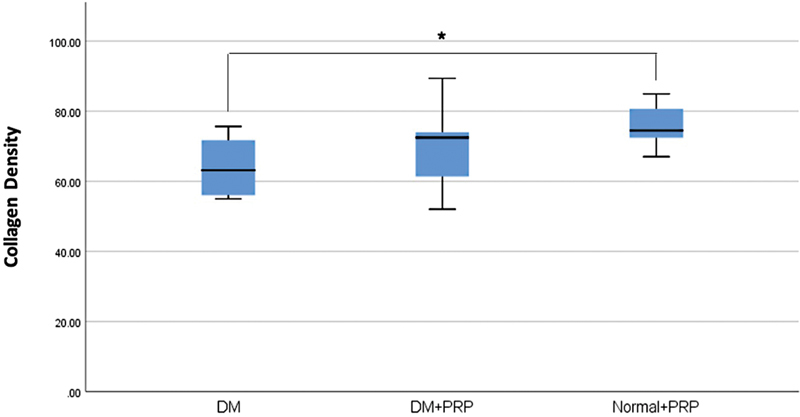
Collagen density of flap tissue in each group (* *p* < 0.05). DM, diabetes mellitus; PRP, platelet-rich plasma.

In both diabetic rat groups, flaps displayed characteristic alterations in collagen fibers. The collagen fibers appeared disorganized compared with the nondiabetic rat group ( Fig. 7 ). Quantification of collagen density revealed an increasing trend in PRP-treated diabetic rats compared with untreated diabetic rats, with median values of 72.48 (IQR 13.72) and 63.13 (IQR 16.91), respectively ( Fig. 8 ). However, at seventh day postoperatively, the difference was not statistically significant ( *p* = 0.198).

## Discussion

The findings of this study demonstrate that the application of PRP injection as flap preconditioning enhances skin flap viability in diabetic conditions. This enhancement reaches a level comparable to nondiabetic rats with PRP injection. This improvement in flap survival could have been mediated by the increase in VEGF levels and subsequent angiogenesis of the flap, as revealed in this study. Similar phenomenon has been observed in previous studies utilizing PRP to aid in skin defect closure. 10 11 Immediately after random skin flap elevation, perfusion pressure of the flap decreases, which then increases the risk of distal flap necrosis. Ischemia–reperfusion injury exacerbates the insult to flap survival due to elevated reactive oxygen species and the inflammatory cascade, leading to vasoconstriction, platelet aggregation, and vessel occlusion. 12

Several studies have revealed a decreased survival of random skin flap and perforator flap in rats with tissue ischemia due to diabetes. 13 Chronic hyperglycemia in diabetes contributes to endothelial cell dysfunction, reduced angiogenesis, and dysregulation of endothelial nitrogen oxide synthase, subsequently disrupting the healing process. Additionally, diabetes leads to increased oxidative stress and proinflammatory cytokines such as TNF-α, IL-6, soluble E-selectin, soluble CD40L, soluble intercellular adhesion molecule, C-reactive protein, and monocyte chemoattractant protein, further complicating flap healing. 14 The use of PRP has been described as an adjunctive therapy with superior results for conditions resulting from microvascular complications in diabetic patients, for instance, in diabetic foot ulcers. 15

The increase of VEGF levels observed in flaps preconditioned with PRP likely played a significant role in enhancing flap vascularization. VEGF functions as a pivotal growth factor for angiogenesis and has been extensively studied, particularly in patients with healing disturbances such as those with diabetes and malignancy. 16 It has been demonstrated that PRP therapy stimulates VEGF release in tissues, along with other cytokines that support soft tissue healing, including platelet-derived growth factor (PDGF), transforming growth factor-β (TGF-β), insulin-like growth factor (IGF), and epidermal growth factor (EGF). 11 Furthermore, VEGF levels in tissue would increase with platelet activation, playing a crucial role in inducing endothelial cell proliferation in the angiogenesis process. Additionally, VEGF exhibits vasodilator effects through signaling pathways mediated by specific cytokines, such as through the Akt-mTOR-HIF-1α (kinase B (Akt), mammalian target of rapamycin (mTOR), and hypoxia-inducible factor 1-alpha (HIF-1a)) activation pathway. 17

While chronic hyperglycemia is known to reduce VEGF production and disrupt its signaling, some studies have demonstrated that serum VEGF concentration may also be elevated in diabetic patients due to prolonged ischemia and reduced oxygen tension, two of the most potent stimuli for VEGF secretion. 18 This may explain the higher VEGF levels and flap viability observed in diabetic rats injected with PRP compared with nondiabetic ones, although it was not statistically significant.

Inactivation of endogenous VEGF expression leads to decreased angiogenesis, further emphasizing the importance of VEGF in this process. 19 Previous research by Kryger et al revealed that administration of exogenous VEGF via various routes of administration protected against hypoxic damage in random skin flaps. 20 PRP also increases levels of PDGF, TGF-β, IGF, EGF, and VEGF, thereby promoting enhanced angiogenesis, endothelial cell migration, and acceleration of tissue regeneration, ultimately improving overall wound healing outcomes in diabetes. 11

The efficacy of tissue reconstruction utilizing flaps relies heavily on adequate vascularization. Cheng et al showed how subcutaneous injection of PRP increased angiogenesis and improved fur growth in rats. 21 Furthermore, it was found that PRP administration alone or in combination with adipose mesenchymal stem cells and endothelial progenitor cells accelerated wound healing in diabetic mice by augmenting angiogenesis through the Notch1 pathway. 22 Moreover, the use of PRP in diabetic foot ulcers also reduces the rate of wound infection. 23

Activation of PRP is known to stimulate the coagulation system and increase the release of growth factors such as PDGF-AA, PDGF-AB, and TGF-β, which have anti-inflammatory properties and contribute to modulating the inflammatory response, thereby promoting optimal wound healing conditions. 24 Consequently, the bioavailability of these inflammatory and regeneration-modulator cytokines increases, and further facilitates their utilization. Although our study utilized activated PRP, several studies have found that activated PRP was not superior to inactivated PRP. 25

In a recent study, Nugroho et al demonstrated that local administration of PRP injection 24 hours prior to random skin flap elevation procedure increased flap viability in Wistar rats. 10 Our findings align with this previous research, indicating that flap preconditioning with subcutaneous injection of PRP 1 day prior to flap procedure increased flap survival by 20.1% compared with those without PRP, even under diabetic conditions. Previous research by Qian et al has also confirmed the effects of PRP in augmenting collagen production and promoting tissue regeneration in diabetic rats. 26 In diabetic conditions, collagen metabolism is disrupted due to hyperglycemia-induced oxidative stress, resulting in accumulation of advanced glycation end products, leading to decreased collagen synthesis and damage to normal collagen structure. 27 Consequently, there is a loss of flexibility and solubility in the collagen triple helix structure, along with an increase in collagen cross-linking. 28 Moreover, dysregulation of matrix metalloproteinase (MMP), involved in the extracellular matrix remodeling process; exacerbates disruption of endothelial cell migration and neoangiogenesis, which are crucial for tissue healing. 29 Theoretically, growth factors released from PRP stimulate dermal fibroblast activation, thereby increasing the expression of procollagen I α 1, elastin, MMP-1, and MMP-2. This phenomenon also increases collagen density in the extracellular matrix remodeling process. 30 In our study, although there was a trend of increasing flap collagen density with PRP administration under diabetic conditions on the seventh day, this difference was not significant. Further research analyzing samples at different phases of wound healing would be necessary, in gain better insight into alterations in collagen synthesis.

Our findings suggest that flap preconditioning with activated PRP injection proved beneficial in improving the viability of modified McFarlane random skin flaps in diabetic rats compared with nontreated diabetic rats. This observation aligns with the increased levels of VEGF and angiogenesis, which significantly contributed to the survival of skin flap tissue. The increase in flap viability, VEGF levels, and angiogenesis in diabetic rats injected with PRP reached levels that were not significantly different from nondiabetic rats receiving PRP injection. Supplementary studies are warranted to further investigate the role of PRP in the remodeling phase of tissue healing in these flaps, as well as to assess the extent of its benefit and safety of its administration in the clinical settings.

Several limitations in our study could be improved in subsequent investigations. First, a control group of nondiabetic rats undergoing flap procedures without PRP preconditioning would have been valuable in estimating the individual effects of each factor studied. Second, the analysis of variables was only performed once, rather than during different phases of the wound healing process.
